# Supplementary material for: Assessment of the Potential Role of Streptomyces in Cave Moonmilk Formation
Source: Front Microbiol. 2017 Jun 29;8:1181. doi: 10.3389/fmicb.2017.01181 (PMC5489568; doi:10.3389/fmicb.2017.01181)
Supplement: Supplementary file 1 [file Table1.DOCX]

***Supplementary Tables***

**Assessment of the Potential Role of *Streptomyces* in Cave Moonmilk Formation**

Marta Maciejewska^1^, Delphine Adam^1^, Aymeric Naômé^1^, Loïc Martinet^1^, Magdalena Całusińska^2^, Philippe Delfosse^2^, Marc Hanikenne^3,4^, Denis Baurain^4,5^, Philippe Compère^6^, Monique Carnol^7^, Hazel Barton^8^, and Sébastien Rigali^1*^

^1^InBioS - Centre for Protein Engineering, Institut de Chimie B6a, University of Liège, B-4000, Liège, Belgium

^2^Environmental Research and Innovation Department, Luxembourg Institute of Science and Technology, Rue du Brill 41, Belvaux, L-4422, Luxembourg

^3^InBioS - Functional Genomics and Plant Molecular Imaging, University of Liège, B-4000 Liège, Belgium

^4^PhytoSYSTEMS, University of Liège, B-4000 Liège, Belgium

^5^InBioS – Eukaryotic Phylogenomics, University of Liège, B-4000, Liège, Belgium

^6^Department of Biology, Ecology and Evolution & Centre of Aid for Research and Education in Microscopy (CAREm-ULg), Institute of Chemistry B6a University of Liège, B-4000, Liège, Belgium

^7^InBioS - Plant and Microbial Ecology, Botany B22, University of Liège, B-4000, Liège, Belgium

^8^Department of Biology, University of Akron, Akron, Ohio, United States of America

^*^Corresponding author. E-mail: srigali@ulg.ac.be; Tel: +32 4 366 98 30; Fax: +32 4 366 33 64

**
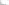

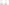
Supplementary Table 1.** List of *Streptomyces coelicolor* genes and number of corresponding orthologous proteins used to generate profile hidden Markov models (HMMs) which were screened against moonmilk *Streptomyces* genomes.

| Gene name | **Gene designations** | **Gene product** | **Nr of orthologs in COG** |
| --- | --- | --- | --- |
| CO_2_ hydration | | | |
| β-CA (1) | SCO3721 | putative carbonic anhydrase (clade A) | 37 |
| β-CA (2) | SCO6055 | probable carbonic anhydrase (clade A) | 32 |
| CA_sulf | SCO6054 | sulfate transporter family protein in cluster with carbonic anhydrase (SulP-type transporter) | 46 |
| Active calcium transport | | | |
| *chaA* | SCO1962 | ionic transporter | 36 |
| NO_3_/NO_2_ transport | | | |
| *narK2* | SCO0213 | possible nitrate/nitrite antiporter protein | 19 |
| *narK* | SCO2959 | probable putative nitrate extrusion protein | 43 |
| NO_3_ assimilation | | | |
| *nasA* | SCO2473 | assimilatory nitrate reductase catalytic subunit | 17 |
| NO_3_ respiration | | | |
| *narG / G2 / G3* | SCO6535 / SCO0216 / SCO4947 | putative nitrate reductase alpha chain NarG | 24 |
| *narH / H2 / H3* | SCO6534 / SCO0217 / SCO4948 | putative nitrate reductase beta chain NarH | 25 |
| *narJ / J3* | SCO6533 / SCO4949 | putative nitrate reductase delta chain NarJ | 30 |
| *narJ2* | SCO0218 | putative nitrate reductase delta chain NarJ2 | 7 |
| *narI / I2 / I3* | SCO6532 / SCO0219 / SCO4950 | putative nitrate reductase gamma chain NarI | 26 |
| NO_2_ reduction | | | |
| *nirB1* | SCO2486 | putative nitrite reductase NirB | 40 |
| *nirB2* | SCO2487 | putative nitrite reductase large subunit NirB | 47 |
| *nirD* | SCO2488 | putative nitrite reductase small subunit NirD | 49 |
| Ureolysis | | | |
| *ureA* | SCO1236 | urease gamma subunit | 53 |
| *ureB* | SCO1235 | urease beta subunit | 52 |
| *ure(AB)* | SCO5525 | fusion of urease beta and gamma subunits | 41 |
| *ureC* | SCO1234 | urease alpha subunit | 45 |
| *ureCb* | SCO5526 | urease alpha subunit | 40 |
| *ureF* | SCO1233 | urease accessory protein | 45 |
| *ureG* | SCO1232 | urease accessory protein | 53 |
| *ureD* | SCO1231 | urease accessory protein | 51 |

**Supplementary Table 2.** List of *Streptomyces* strains used to generate clusters of orthologous groups of proteins (COGs) and their assembly accession codes.

| **Strain** | **RefSeq assembly accession** |
| --- | --- |
| *Streptomyces* sp. CNQ-509 | GCF_001011035.1 |
| *Streptomyces* *incarnatus* NRRL 8089 | GCA_001027185.1^a^ |
| *Streptomyces* sp. CFMR 7 CFMR-7 | GCF_001278095.1 |
| *Streptomyces* *venezuelae* ATCC 15439 | GCF_001406115.1 |
| *Streptomyces* sp. CdTB01 | GCF_001484565.1 |
| *Streptomyces* sp. 4F | GCF_001484705.1 |
| *Streptomyces* *collinus* Tu 365 | GCF_000444875.1 |
| *Streptomyces* sp. PVA 94-07 | GCF_000495755.1 |
| *Streptomyces* sp. GBA 94-10 | GCF_000495635.1 |
| *Streptomyces* *davawensis* JCM 4913 | GCF_000349325.1 |
| *Streptomyces* *albulus* NK660 | GCF_000695235.1 |
| *Streptomyces* sp. PAMC 26508 | GCF_000364805.1 |
| *Streptomyces* sp. 769 | GCF_000816025.1 |
| *Streptomyces* *anulatus* ATCC 11523 | GCF_001434355.1 |
| *Streptomyces* *rapamycinicus* NRRL 5491 | GCA_000418455.1^a^ |
| *Streptomyces* sp. Mg1 | GCF_000412265.2 |
| *Streptomyces* *niveus* NCIMB 11891 | GCF_000497425.1 |
| *Streptomyces* *roseochromogenus* *subsp. oscitans* DS 12.976 | GCF_000497445.1 |
| *Streptomyces* sp. CCM_MD2014 | GCF_000772045.1 |
| *Streptomyces* sp. PBH53 | GCA_001040905.1 ^a^ |
| *Streptomyces* sp. SirexAA-E | GCF_000177195.2 |
| *Streptomyces* *ambofaciens* ATCC 23877 | GCF_001267885.1 |
| *Streptomyces* *avermitilis* MA-4680 = NBRC 14893 | GCF_000968255.1^b^ |
| *Streptomyces* *albulus* ZPM | GCF_000963515.1 |
| *Streptomyces* *bingchenggensis* BCW-1 | GCF_000092385.1 |
| *Streptomyces* *scabiei* 87.22 | GCF_000091305.1 |
| *Streptomyces* *cattleya* NRRL 8057 = DSM 46488 | GCF_000240165.1 |
| *Streptomyces* *clavuligerus* ATCC 27064 | GCF_000163875.1 |
| *Streptomyces* *coelicolor* A3(2) | GCF_000203835.1 |
| *Streptomyces* *pratensis* ATCC 33331 | GCF_000176115.2 |
| *Streptomyces* *fulvissimus* DSM 40593 | GCF_000385945.1 |
| *Streptomyces* *glaucescens* GLA.O | GCF_000761215.1 |
| *Streptomyces* *griseus subsp. griseus* NBRC 13350 | GCF_000010605.1 |
| *Streptomyces* *hygroscopicus subsp. jinggangensis* TL01 | GCF_000340845.1 |
| *Streptomyces* *hygroscopicus subsp. jinggangensis* 5008 | GCF_000245355.1 |
| *Streptomyces* *hygroscopicus subsp. limoneus* KCTC 1717 | GCF_001447075.1 |
| *Streptomyces* *leeuwenhoekii* C34 = DSM 42122 = NRRL B-24963 | GCF_001013905.1 |
| *Streptomyces* *lividans* 1326 | GCF_000403665.1 |
| *Streptomyces* *lividans* TK24 | GCF_000403665.1 |
| *Streptomyces* *albus* DSM 41398 | GCF_000827005.1 |
| *Streptomyces* *nodosus* ATCC 14899 | GCF_000819545.1 |
| *Streptomyces* *pristinaespiralis* HCCB 10218 | GCF_001278075.1 |
| *Streptomyces* *pristinaespiralis* ATCC 25486 | GCF_000154945.1 |
| *Streptomyces* *sviceus* ATCC 29083 | GCF_000154965.1 |
| *Streptomyces* *violaceusniger* Tu 4113 | GCF_000147815.2 |
| *Streptomyces* sp. Tu6071 | GCF_000213055.1 |
| *Streptomyces* *venezuelae* ATCC 10712 | GCF_000253235.1 |
| *Streptomyces* *vietnamensis* GIM4.0001 | GCF_000830005.1 |
| *Streptomyces* *xiamenensis* MCCC 1A01550 | GCF_000993785.2 |
| *Streptomyces* *lydicus* A02 | GCF_000952035.1 |
| *Streptomyces* *cyaneogriseus subsp. noncyanogenus* NMWT 1 | GCF_000931445.1 |
| *Streptomyces* *globisporus* C-1027 | GCF_000261345.2 |
| *Streptomyces* *albus* J1074 | GCF_000359525.1 |
| *Streptomyces* *reticuli* TUE45 | GCF_001511815.1 |

^a^ GenBank assembly accession. No RefSeq accession because the assembly is missing rRNA genes.

^b^ Included despite “scaffold” assembly level. Complete genome published on 2016/03/24 with accession GCF_000009765.2.

**Supplementary Table 3.** Accession numbers of biomineralization-related genes derived from moonmilk *Streptomyces* genomes.

|  | **β -CA(1)** | **β -CA(2)** | **CA-Sulf** | ***chaA*** | ***narK2*** | ***narK*** | ***nasA*** | ***narG*** | ***narH*** | ***narJ*** | ***narJ2*** | ***narI*** |
| --- | --- | --- | --- | --- | --- | --- | --- | --- | --- | --- | --- | --- |
| **MM48** | KY288441  KY288442  KY288443  KY288444  KY288445 | KY288389 | KY288320 | KY288341 | KY288264 | KY288293 |  | KY288360 | KY288360 | KY288360 |  | KY288360 |
| **MM17** | KY288423  KY288424  KY288425  KY288426  KY288427  KY288428 | KY288380 | KY288312 | KY288338 | KY288256 | KY288285 | KY288189 | KY288348 |  |  |  |  |
| **MM6** | KY288454  KY288455  KY288456  KY288457 |  | KY288324 | KY288346 | KY288268 | KY288297 | KY288202 |  |  |  |  |  |
| **MM104** | KY288399  KY288400 | KY288361  KY288362  KY288363 | KY288300 | KY288327 | KY288242 | KY288271 |  |  |  |  |  |  |
| **MM1** | KY288430  KY288431 | KY288382 | KY288314 |  | KY288258 | KY288287 | KY288191 |  |  |  |  |  |
| **MM24** | KY288435  KY288436 | KY288385  KY288386 | KY288317 | KY288339 | KY288261 | KY288290 | KY288194 |  |  |  |  |  |
| **MM12** | KY288418  KY288419 | KY288375  KY288376 |  | KY288336 | KY288253 | KY288282 | KY288186 |  |  |  |  |  |
| **MM100** |  |  |  |  |  |  |  |  |  |  |  |  |
| **MM117** | KY288414 | KY288369 | KY288308 |  | KY288250 | KY288279 |  |  |  |  |  |  |
| **MM122** | KY288415 | KY288370  KY288371  KY288372 |  |  | KY288251 | KY288280 | KY288184 |  |  |  |  |  |
| **MM99** | KY288459 | KY288398 | KY288326 |  | KY288270 | KY288299 | KY288204 |  |  |  |  |  |
| **MM14** | KY288422 | KY288378  KY288379 | KY288311 |  | KY288255 | KY288284 | KY288188 |  | KY288465 |  | KY288465 | KY288465 |
| **MM105** | KY288401 |  | KY288301 | KY288328 | KY288243 | KY288272 | KY288175 |  |  |  |  |  |
| **MM111** | KY288413 | KY288368 | KY288307 | KY288334 | KY288249 | KY288278 | KY288181 | KY288355 | KY288355 |  | KY288355 | KY288355 |
| **MM108** | KY288406  KY288407  KY288408 | KY288366 | KY288304 | KY288332 | KY288246 | KY288275 |  |  |  |  |  |  |
| **MM106** | KY288402 |  | KY288302 | KY288329  KY288330 | KY288244 | KY288273 | KY288176 | KY288350  KY288351  KY288352 | KY288350  KY288351  KY288352 |  | KY288350  KY288351  KY288352 | KY288350  KY288351  KY288352 |
| **MM128** | KY288416  KY288417 | KY288373  KY288374 | KY288309 | KY288335 | KY288252 | KY288281 | KY288185 |  |  |  |  |  |
| **MM10** | KY288411  KY288412 | KY288367 | KY288306 | KY288333 | KY288248 | KY288277 | KY288180 | KY288349  KY288354  KY288357  KY288358  KY288359 | KY288354  KY288357  KY288358  KY288359 | KY288357  KY288358  KY288359 | KY288354 | KY288354  KY288357  KY288358  KY288359 |
| **MM13** | KY288420  KY288421 | KY288377 | KY288310 | KY288337 | KY288254 | KY288283 | KY288187 | KY288347 |  |  |  | KY288464 |
| **MM68** | KY288452  KY288453 | KY288395  KY288396 | KY288323 | KY288344  KY288345 | KY288267 | KY288296 | KY288201 |  |  |  |  |  |
| **MM59** | KY288446  KY288447  KY288448 | KY288390  KY288391 | KY288321 | KY288342 | KY288265 | KY288294 | KY288199 |  |  |  |  |  |
| **MM5** | KY288449  KY288450  KY288451 | KY288392  KY288393  KY288394 | KY288322 | KY288343 | KY288266 | KY288295 | KY288200 |  |  |  |  |  |
| **MM107** | KY288403  KY288404  KY288405 | KY288364  KY288365 | KY288303 | KY288331 | KY288245 | KY288274 |  |  |  |  |  |  |
| **MM109** | KY288409  KY288410 |  | KY288305 |  | KY288247 | KY288276 | KY288179 | KY288353  KY288468 | KY288353 |  | KY288353 | KY288353  KY288468 |
| **MM19** | KY288429 | KY288381 | KY288313 |  | KY288257 | KY288286 | KY288190 |  |  |  |  |  |
| **MM7** | KY288458 | KY288397 | KY288325 |  | KY288269 | KY288298 | KY288203 | KY288356 | KY288356 |  | KY288356 | KY288356 |
| **MM44** | KY288439  KY288440 | KY288388 | KY288319 |  | KY288263 | KY288292 | KY288196 |  |  |  |  |  |
| **MM21** | KY288432  KY288433 | KY288383 | KY288315 |  | KY288259 | KY288288 | KY288192 |  |  |  |  |  |
| **MM23** | KY288434 | KY288384 | KY288316 |  | KY288260 | KY288289 | KY288193 |  |  |  |  |  |
| **MM3** | KY288437  KY288438 | KY288387 | KY288318 | KY288340 | KY288262 | KY288291 | KY288195 |  |  |  |  |  |

|  | ***nirB1*** | ***nirB2*** | ***nirD*** | ***ureA*** | ***ureB*** | ***ure(AB)*** | ***ureC*** | ***ureF*** | ***ureG*** | ***ureD*** |
| --- | --- | --- | --- | --- | --- | --- | --- | --- | --- | --- |
| **MM48** | KY288233 | KY288233  KY288239 | KY288233 | KY288157  KY288158  KY288159  KY288160  KY288161 | KY288157  KY288158  KY288159  KY288160  KY288161 | KY288097  KY288098 | KY288097  KY288098  KY288157  KY288158  KY288159  KY288160  KY288161 | KY288157  KY288158  KY288159  KY288160  KY288161 | KY288157  KY288158  KY288159  KY288160  KY288161 | KY288157  KY288158  KY288159  KY288160  KY288161 |
| **MM17** | KY288224 | KY288224 | KY288224 | KY288145  KY288466 | KY288145  KY288466 | KY288086  KY288117 | KY288086  KY288117  KY288145 | KY288117  KY288145 | KY288117  KY288145 | KY288117  KY288145 |
| **MM6** | KY288237 | KY288237  KY288461 | KY288237  KY288461 | KY288168 | KY288168 | KY288099 | KY288099  KY288106  KY288168 | KY288168 | KY288168 | KY288168 |
| **MM104** | KY288206  KY288207 | KY288206  KY288207 | KY288206  KY288207 | KY288125 | KY288125 |  | KY288125 | KY288125 | KY288125 | KY288125 |
| **MM1** | KY288226 | KY288226 | KY288226 | KY288147 | KY288147 | KY288088  KY288089  KY288090 | KY288088  KY288089  KY288090  KY288147 | KY288147 | KY288147 | KY288107  KY288147 |
| **MM24** | KY288229  KY288230 | KY288229  KY288230 | KY288229  KY288230 | KY288150  KY288151  KY288152  KY288153  KY288154 | KY288150  KY288151  KY288152  KY288153  KY288154 | KY288093  KY288094 | KY288093  KY288094  KY288150  KY288151  KY288152  KY288153  KY288154 | KY288150  KY288151  KY288152  KY288153  KY288154 | KY288150  KY288151  KY288152  KY288153  KY288154 | KY288109  KY288150  KY288151  KY288152  KY288153  KY288154 |
| **MM12** | KY288220 | KY288220 | KY288220 | KY288141 | KY288141 | KY288084  KY288114 | KY288084  KY288114  KY288141 | KY288114  KY288141  KY288462  KY288463 | KY288114  KY288141  KY288462  KY288463 | KY288114  KY288141  KY288462  KY288463 |
| **MM100** | KY288205 | KY288205 | KY288205 | KY288124 | KY288124 | KY288071  KY288110 | KY288071  KY288102  KY288110  KY288124 | KY288110  KY288124 | KY288110  KY288124 | KY288110  KY288124 |
| **MM117** | KY288215 | KY288215 | KY288215 | KY288136 | KY288136 | KY288081 | KY288081  KY288136 | KY288136 | KY288136 | KY288136 |
| **MM122** | KY288216  KY288217  KY288218 | KY288216  KY288217  KY288218 | KY288216  KY288217  KY288218 | KY288137  KY288138 | KY288137  KY288138 | KY288082  KY288113 | KY288082  KY288113  KY288137  KY288138 | KY288113  KY288137  KY288138 | KY288113  KY288137  KY288138 | KY288113  KY288137  KY288138 |
| **MM99** |  |  |  | KY288171  KY288172  KY288173  KY288174 | KY288171  KY288172  KY288173  KY288174 | KY288101 | KY288101  KY288171  KY288172  KY288173  KY288174 | KY288171  KY288172  KY288173  KY288174 | KY288171  KY288172  KY288173  KY288174 | KY288171  KY288172  KY288173  KY288174 |
| **MM14** | KY288222  KY288223 | KY288222  KY288223 | KY288222  KY288223 | KY288144 | KY288144 | KY288085  KY288116 | KY288085  KY288116  KY288144 | KY288116  KY288144 | KY288116  KY288144 | KY288116  KY288144 |
| **MM105** | KY288208  KY288209 | KY288208  KY288209 | KY288208  KY288209 | KY288126 | KY288126 | KY288072  KY288073  KY288074 | KY288072  KY288073  KY288074  KY288126 | KY288126 | KY288126 | KY288126 |
| **MM111** | KY288214 | KY288214 | KY288214 | KY288134  KY288135 | KY288134  KY288135 | KY288079  KY288080 | KY288079  KY288080  KY288134  KY288135 | KY288134  KY288135 | KY288134  KY288135 | KY288134  KY288135 |
| **MM108** | KY288211 | KY288211 | KY288211 | KY288130 | KY288130 | KY288075  KY288076  KY288077 | KY288075  KY288076  KY288077  KY288130 | KY288130 | KY288130 | KY288130 |
| **MM106** | KY288210 | KY288210 | KY288210 | KY288127 | KY288127 |  | KY288127 | KY288127 | KY288127 | KY288127 |
| **MM128** | KY288219 | KY288219 | KY288219 | KY288139  KY288140 | KY288139  KY288140 | KY288083 | KY288083  KY288139  KY288140 | KY288139  KY288140 | KY288139  KY288140 | KY288139  KY288140 |
| **MM10** | KY288213 | KY288213 | KY288213 | KY288132  KY288133 | KY288132  KY288133 | KY288112 | KY288112  KY288132  KY288133 | KY288112  KY288132  KY288133 | KY288112  KY288132  KY288133 | KY288112  KY288132  KY288133 |
| **MM13** | KY288221 | KY288221 | KY288221 | KY288142  KY288143 | KY288142  KY288143 | KY288115 | KY288115  KY288142  KY288143 | KY288115  KY288142  KY288143 | KY288115  KY288142  KY288143 | KY288115  KY288142  KY288143 |
| **MM68** | KY288236 | KY288236 | KY288236 | KY288166  KY288167 | KY288166  KY288167 | KY288123 | KY288123  KY288166  KY288167 | KY288123  KY288166  KY288167  KY288467 | KY288123  KY288166  KY288167 | KY288123  KY288166  KY288167 |
| **MM59** | KY288234 | KY288234 | KY288234 | KY288162  KY288163 | KY288162  KY288163 | | KY288162  KY288163 | KY288162  KY288163 | KY288162  KY288163 | KY288162  KY288163 |
| **MM5** | KY288235 | KY288235  KY288240  KY288241 | KY288235 | KY288164  KY288165 | KY288164  KY288165 | KY288122 | KY288105  KY288122  KY288164  KY288165 | KY288122  KY288164  KY288165 | KY288122  KY288164  KY288165 | KY288122  KY288164  KY288165 |
| **MM107** |  |  |  |  |  |  | KY288103 |  |  |  |
| **MM109** | KY288212 | KY288212 | KY288212 | KY288131 | KY288131 | KY288078  KY288111 | KY288078  KY288111  KY288131 | KY288111  KY288131 | KY288111  KY288131 | KY288111  KY288131 |
| **MM19** | KY288225 | KY288225 | KY288225 | KY288146 | KY288146 | KY288087 | KY288087  KY288146 | KY288146 | KY288146 | KY288146 |
| **MM7** | KY288238 | KY288238 | KY288238 | KY288169  KY288170  KY288460 | KY288169  KY288170 | KY288100 | KY288100  KY288169  KY288170 | KY288169  KY288170 | KY288169  KY288170 | KY288169  KY288170 |
| **MM44** | KY288232 | KY288232 | KY288232 | KY288156 | KY288156 | KY288096  KY288121 | KY288096  KY288121  KY288156 | KY288121  KY288156 | KY288121  KY288156 | KY288121  KY288156 |
| **MM21** | KY288227 | KY288227 | KY288227 | KY288148 | KY288148 | KY288091  KY288118 | KY288091  KY288118  KY288148 | KY288118  KY288148 | KY288118  KY288148 | KY288118  KY288148 |
| **MM23** | KY288228 | KY288228 | KY288228 | KY288149 | KY288149 | KY288092  KY288119 | KY288092  KY288119  KY288149 | KY288119  KY288149 | KY288119  KY288149 | KY288119  KY288149 |
| **MM3** | KY288231 | KY288231 | KY288231 | KY288155 | KY288155 | KY288095  KY288120 | KY288095  KY288104  KY288120  KY288155 | KY288120  KY288155 | KY288120  KY288155 | KY288108  KY288120  KY288155 |
