# Supplementary material for: Assessment of the Potential Role of Streptomyces in Cave Moonmilk Formation
Source: Front Microbiol. 2017 Jun 29;8:1181. doi: 10.3389/fmicb.2017.01181 (PMC5489568; doi:10.3389/fmicb.2017.01181)

Supplementary Figures

Assessment of the Potential Role of *Streptomyces* in Cave Moonmilk Formation

Marta Maciejewska[1], Delphine Adam[1], Aymeric Naômé[1], Loïc Martinet[1], Magdalena Całusińska[2], Philippe Delfosse[2], Marc Hanikenne[3,4], Denis Baurain[4,5], Philippe Compère[6], Monique Carnol[7], Hazel Barton[8], and Sébastien Rigali[1*]

[1]InBioS - Centre for Protein Engineering, Institut de Chimie B6a, University of Liège, B-4000, Liège, Belgium
[2]Environmental Research and Innovation Department, Luxembourg Institute of Science and Technology, Rue du Brill 41, Belvaux, L-4422, Luxembourg
[3]InBioS - Functional Genomics and Plant Molecular Imaging, University of Liège, B-4000 Liège, Belgium
[4]PhytoSYSTEMS, University of Liège, B-4000 Liège, Belgium
[5]InBioS – Eukaryotic Phylogenomics, University of Liège, B-4000, Liège, Belgium
[6]Department of Biology, Ecology and Evolution & Centre of Aid for Research and Education in Microscopy (CAREm-ULg), Institute of Chemistry B6a University of Liège, B-4000, Liège, Belgium
[7]InBioS - Plant and Microbial Ecology, Botany B22, University of Liège, B-4000, Liège, Belgium
[8]Department of Biology, University of Akron, Akron, Ohio, United States of America
[*]Corresponding author. E-mail: srigali@ulg.ac.be; Tel: +32 4 366 98 30; Fax: +32 4 366 33 64

Supplementary Figure 1. Location of the Grotte de Collemboles (Springtails' Cave) in Comblain-au-Pont in Belgium, together with the map of the cave and visualization of moonmilk deposits from sampling sites used in this study (COL1, COL3, COL4) (figure taken from Maciejewska et al. 2016).

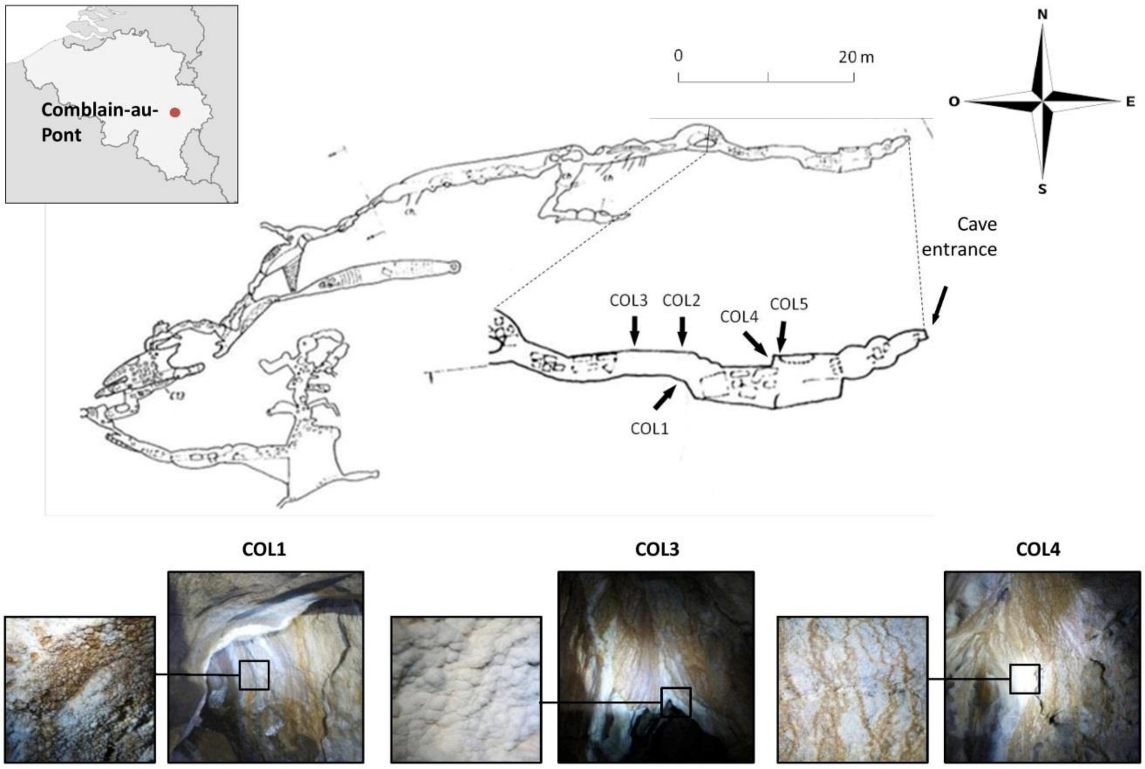

Cave description: The Grotte des Collemboles is a 70 m long and 20 m deep fissure cave, with an annual temperature oscillating around 11.5°C. The access information and location details are restricted due to the cave protection policies. Above the cave there is a deciduous wood at proximity to crop and breeding fields. White to brownish-orange moonmilk deposits are found on the walls and the ceilings within the first 20 m from the cave entrance, and associated with percolating waters.

Supplementary Figure 2. Multilocus sequence analysis (MLSA) of moonmilk derived strains from Grotte des Collemboles in Belgium and phenotype of the representative strains (figure taken from Maciejewska et al. 2016). **A)** Representative strain from each of 30 phylotypes (indicated in brackets) used for metabolic screening is indicated in bold. **B)** Phenotype of representative strains of each phylotype (MLSA established phylotype is indicated in brackets together with its corresponding 16S rRNA-based phylotype) (Maciejewska et al. 2016).

A

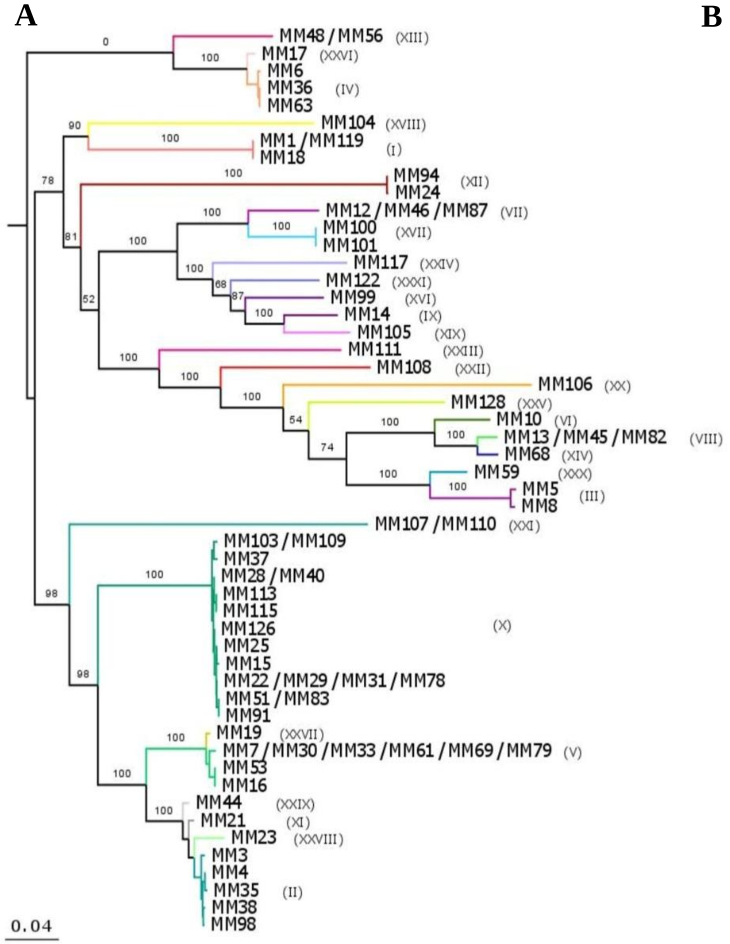

B

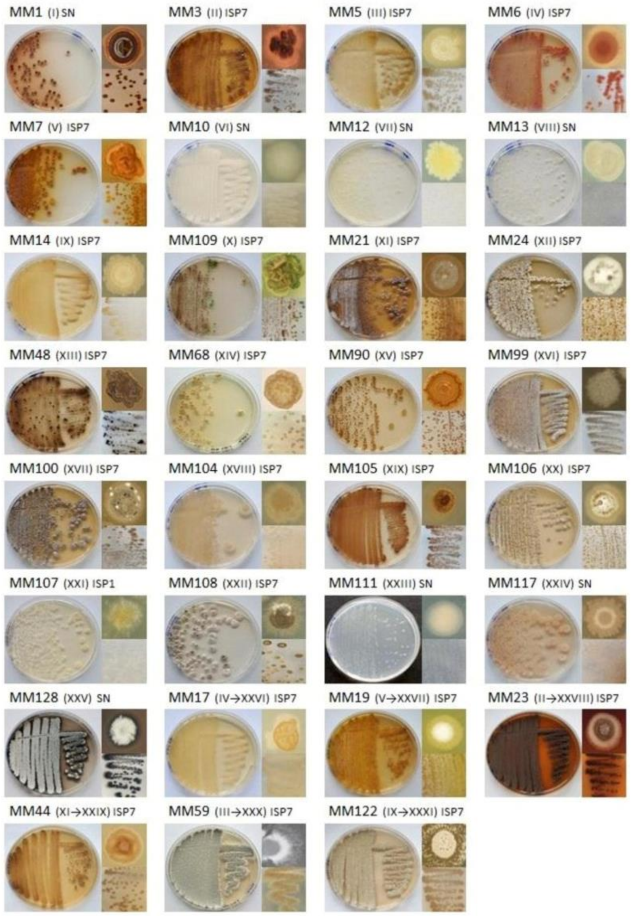

Supplementary Figure 3. EDS spectra of needle-fiber crystals (A), and nano-sized filaments (B) present at the surface of an ethanol-fixed moonmilk speleothem together with the corresponding SEM image (spectra locations). A recurrent higher carbon content in nano-sized filaments than in needle-fiber crystals suggests the possible organic origin of the nano-sized filaments.

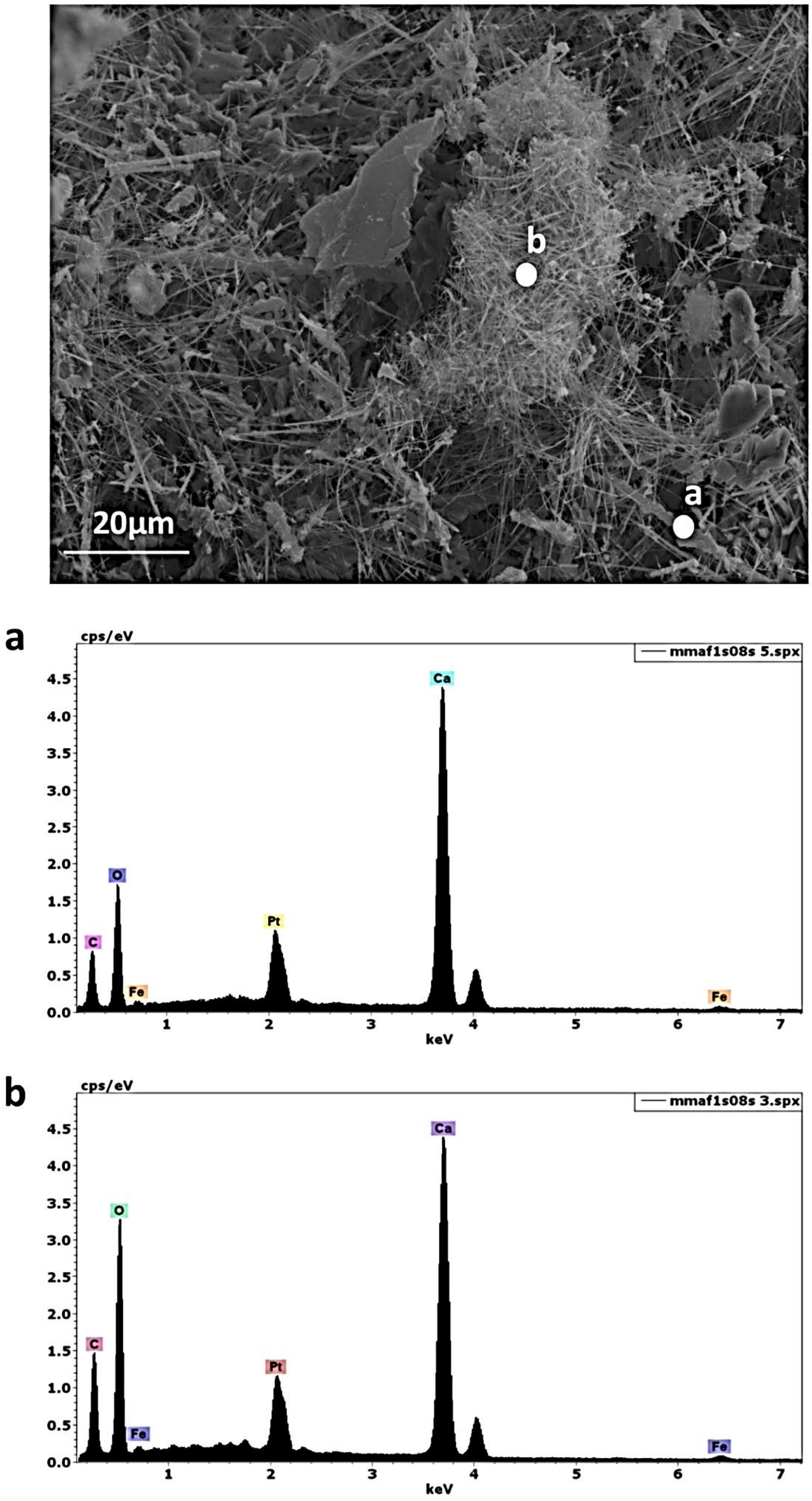

Supplementary Figure 4. Speleogenesis-related metabolic activities of moonmilk isolates. A) urea degradation to ammonia, **B**) ammonification of peptides/amino acids, **C**) nitrate reduction, and **D**) nitrite reduction. The performance of investigated activity is assigned with the following symbols: +++, strong; ++ good; +, moderate; +/-, weak; -, no activity. For nitrate and nitrite reductions assays the results performed in solid media are shown on the top and in liquid media on the bottom for each strain. Abbreviations: MM – moonmilk isolate, UAB - Christensen's Urea Agar Base, EC - *Escherichia coli* ATCC 25922, KP - *Klebsiella pneumoniae* ATCC 13883, NA – nutrient agar, CF - *Citrobacter freundii* ATCC 43864, PA - *Pseudomonas aeruginosa* ATCC 27853.

A) Urea degradation

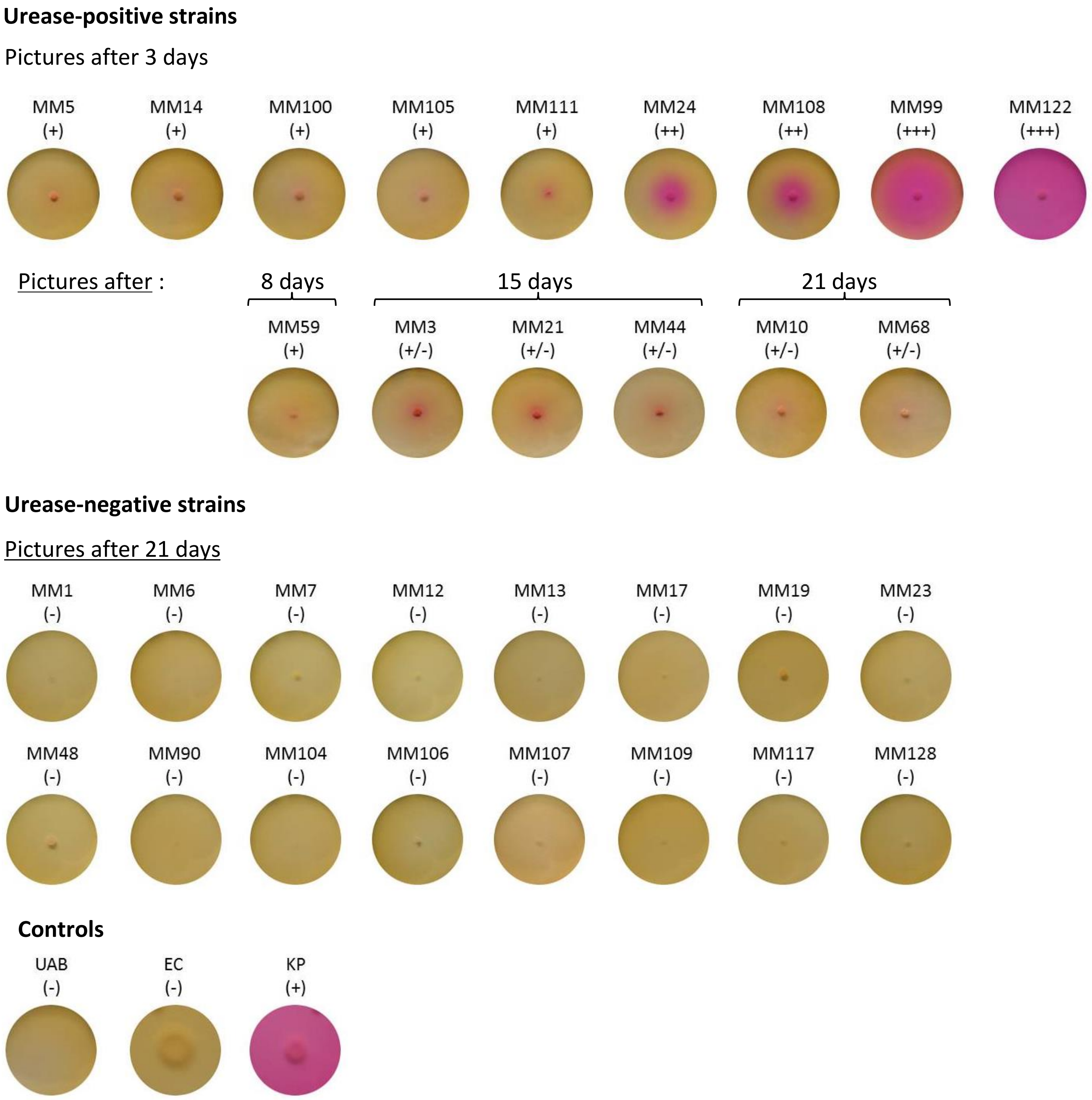

B) Ammonification of peptides/amino acids

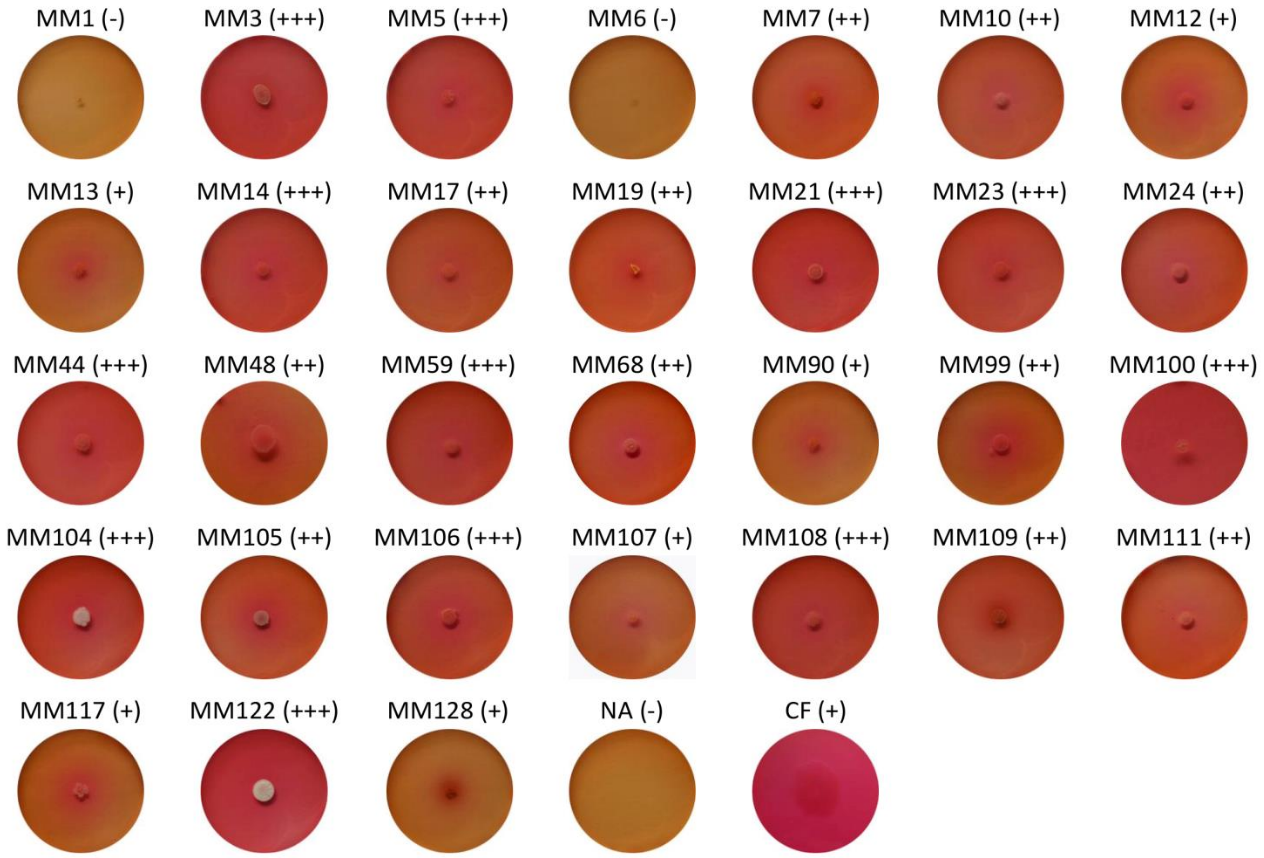

C) Nitrate reduction

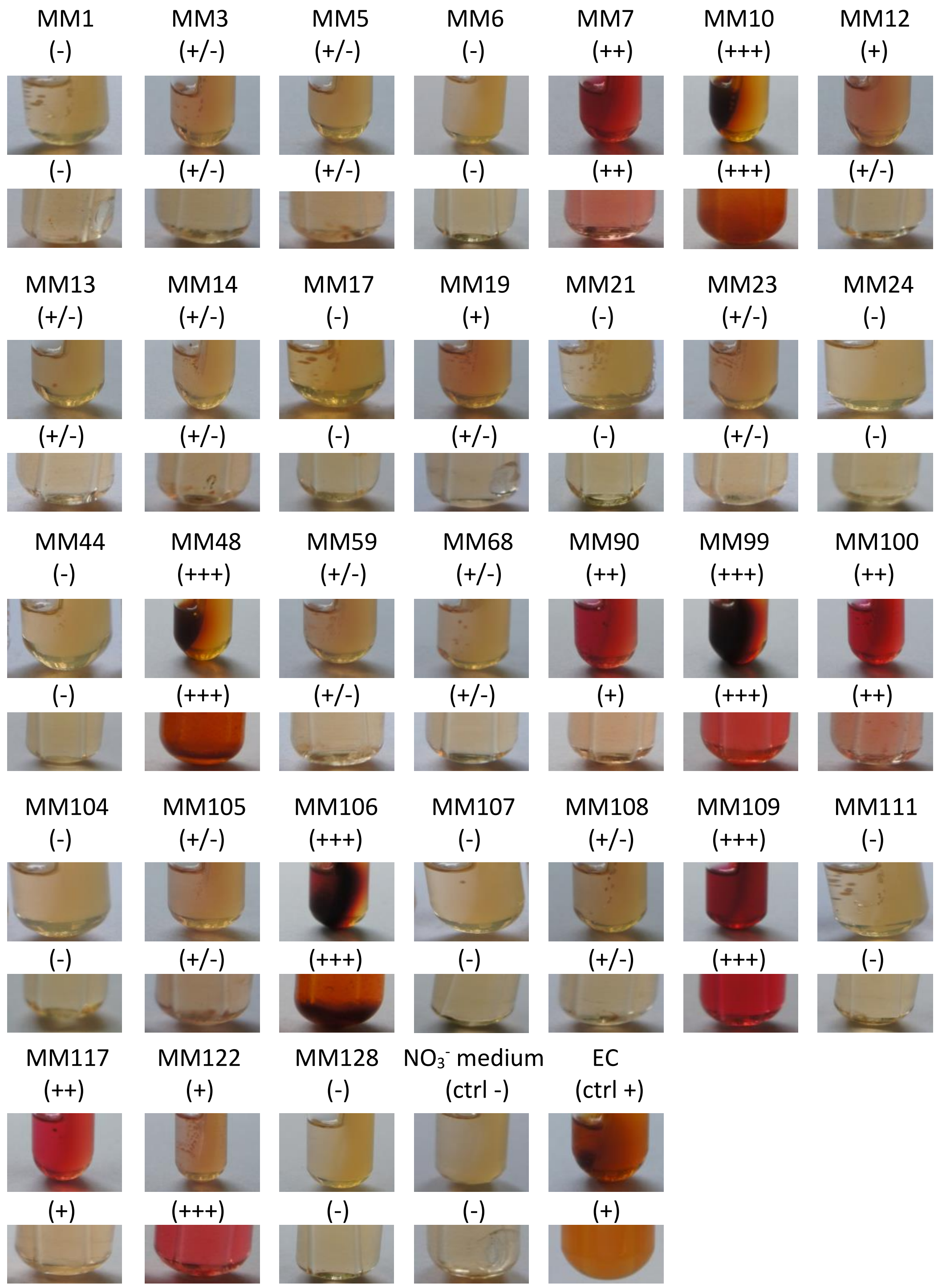

D) Nitrite reduction

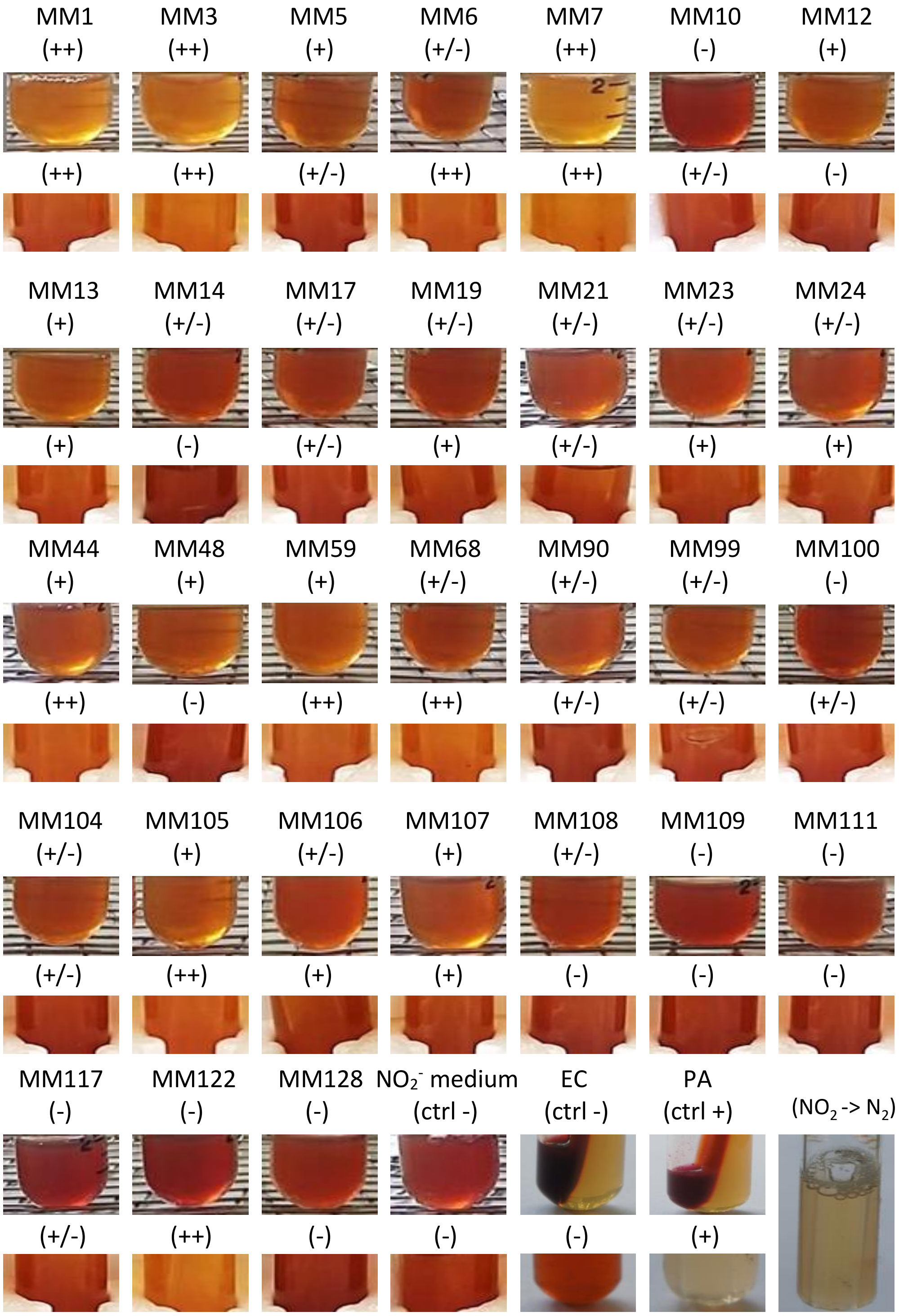

Supplementary Figure 5. LV-SEM-BSE images (A-D) and position beam EDS spectra (1-8) of calcareous deposits produced by isolate MM99 grown in CPA (A) and B-4 (B) and MM24 grown in CPA (C) and B-4 (D) media together with quantitative data for the main elements (E). The major C (Kα at 0.277 keV), O (Kα at 0.524 keV) and Ca (Kα at 3,690 keV) peaks are always associated to the spherulitic deposits, confirming their calcareous composition. While C and O are also present in the bacterial biomass, which is characterized by the presence of the minor elements - N (Kα peak at 0.392 keV), P (Kα peak at 2.013 keV), S (Kα peak at 2.307 keV) as well as K (Kα peak at 3.312 keV). Na (Kα peak at 1.041 keV) and Cl (Kα peak at 2.621 keV) were attributed to the culture medium, while Si (Kα peak at 1.739 keV) may come from the supporting glass slide. The table (E) shows the semi-quantitative atomic percentages directly provided by the ZAF method form spectra. Due to the roughness of the samples, they are only indicatives of the elemental concentrations that produced the dominant peaks. At the same time, they support the calcareous composition of the deposits where the C, O and Ca atomic percent (at.%) were very roughly those found in the $CaCO_3$ stoichiometry.

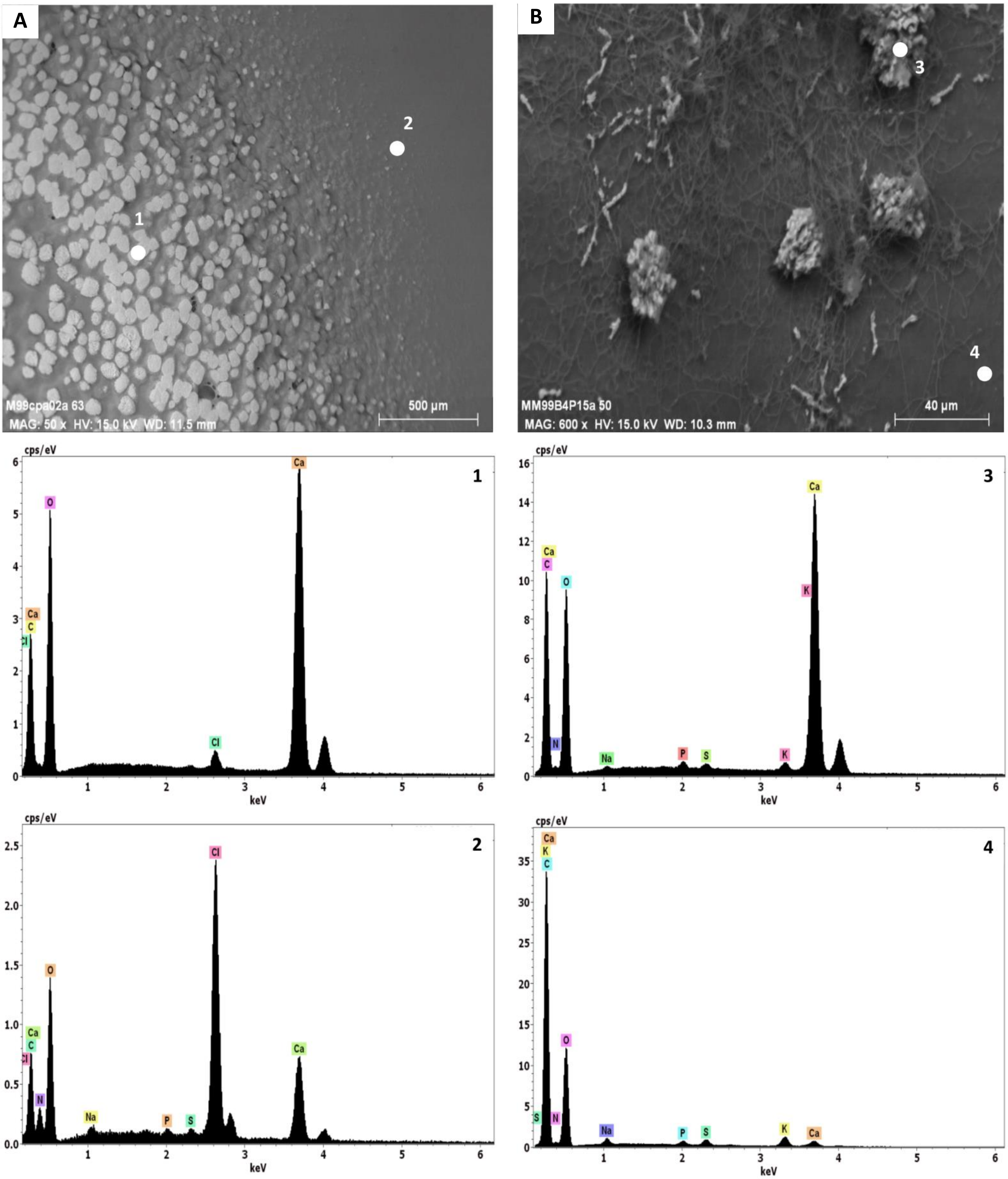
A
B
1
2
3
4
M99cpa02a 63
MAG: 50 x HV: 15.0 kV WD: 11.5 mm
500 µm
MM99B4P15a 50
MAG: 600 x HV: 15.0 kV WD: 10.3 mm
40 µm
cps/eV
keV
Ca
O
C
Cl
N
Na
P
S
K

frontiers

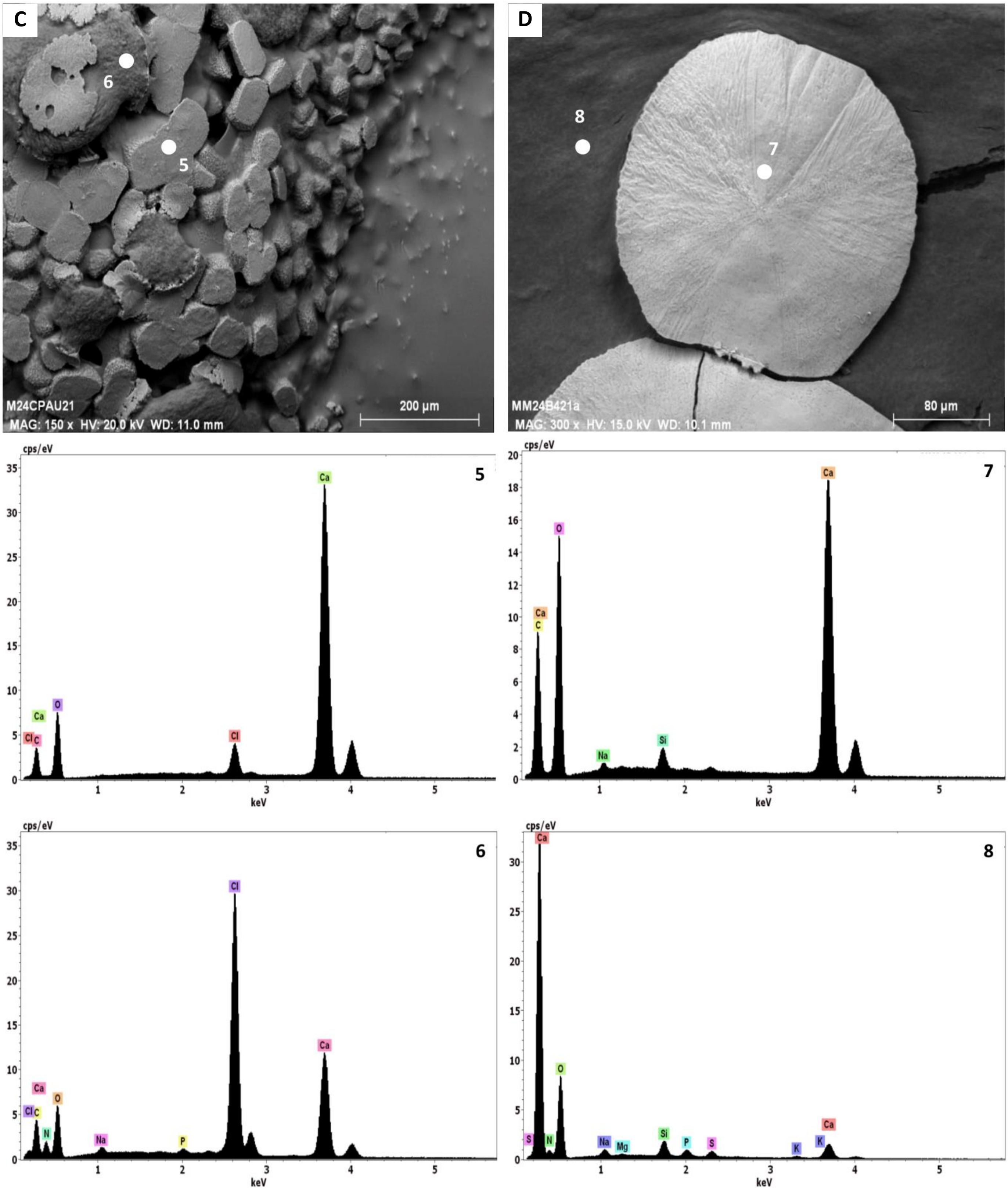
C
D
6
5
8
7
M24CPAU21
MAG: 150 x HV: 20.0 kV WD: 11.0 mm
200 µm
MM24B421a
MAG: 300 x HV: 15.0 kV WD: 10.1 mm
80 µm
cps/eV
keV
Ca
O
Cl
C
Na
Si
N
P
S
Mg
K

E)

	[norm. at.%]							
	MM99				MM24			
	CPA		B-4		CPA		B-4	
**Element**	**Mineral (1)**	**Biomass (2)**	**Mineral (3)**	**Biomass (4)**	**Mineral (5)**	**Biomass (6)**	**Mineral (7)**	**Biomass (8)**
**C**	24.44	32.01	27.64	59.86	19.55	33.23	26.06	65.56
**N**	-	19.00	2.58	-	-	22.60	-	0.45
**O**	60.29	36.93	53.76	37.94	57.85	30.20	58.34	30.93
**Na**	-	0.32	0.19	0.38	-	0.27	0.59	0.33
**Si**	-	-	-	-	-	-	0.71	0.64
**P**	-	0.12	0.20	0.19	-	0.10	-	0.29
**S**	-	0.18	0.15	0.28	-		-	0.26
**Cl**	0.62	7.71	-	-	1.68	8.17	-	
**K**	-	-	0.32	0.78	-	-	-	0.13
**Ca**	14.64	3.73	15.16	0.57	20.92	5.43	14.30	1.37

Supplementary Figure 6. Oxidative reduction of glucose on Hugh and Leifson's OF basal medium (OF) by moonmilk *Streptomyces* phylotypes. *Pseudomonas aeruginosa* ATCC 27853 was used as a positive control. The strength of media acidification visible as change of color from blue to yellowish/transparent is expressed with the following symbols: +++, strong; ++ good; +, moderate; +/-, weak; -, no acidification.

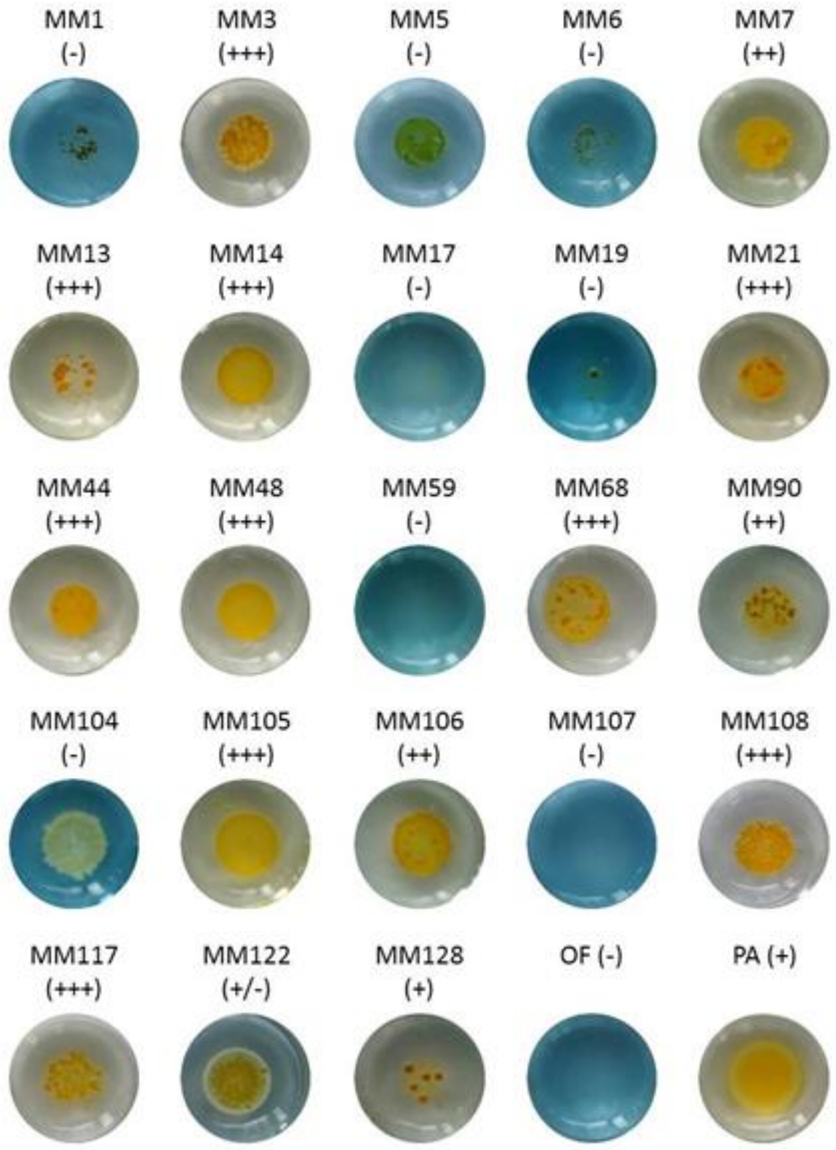

Supplement: Supplementary file 2 [file Image1.PDF]
